# Real-World Evidence of Efficacy and Safety of Levonadifloxacin (Oral and IV) in the Management of Acute Bacterial Skin and Skin Structure Infections (ABSSSI): Findings of a Retrospective, Multi-Center Study

**DOI:** 10.7759/cureus.24299

**Published:** 2022-04-20

**Authors:** Kapil D Mehta, Jai B Sharma, Ashok Anand, Pavan K Reddy N, Pramod Kadam, Khokan Debnath, Sandeep Bhapkar, Bini M Thampi

**Affiliations:** 1 Medical Affairs, Wockhardt Ltd., Mumbai, IND; 2 Medical Oncology, Action Cancer Hospital, New Delhi, IND; 3 Critical Care and Emergency Medicine, Sir Ganga Ram City Hospital (SGRH), New Delhi, IND; 4 Critical Care Medicine, CARE Hospitals, Hyderabad, IND; 5 Surgery, Ruby Hall, Pune, IND; 6 Medical Affairs, Clinical Operations, Pharmacovigilance (PV) and Regulatory, Wockhardt Ltd., Mumbai, IND

**Keywords:** microbial success, clinical success, alalevonadifloxacin, levonadifloxacin, absssi

## Abstract

Background

Antimicrobial resistance by bacteria poses a substantial threat to the success in the treatment of acute bacterial skin and skin structure infections (ABSSSI). Levonadifloxacin is a novel benzoquinolizine subclass of quinolone which has a broad spectrum of activity, available in both oral and intravenous formulations for the treatment of skin structure infections caused by Gram-positive pathogens including methicillin-resistant Staphylococcus aureus (MRSA).

Patients and methods

This prescription event monitoring study captured data of 227 patients receiving levonadifloxacin (oral and/or IV) in a real-world setting to assess the safety and efficacy in the treatment of ABSSSI. Study outcomes were a clinical and microbial success at the end of therapy and safety was assessed based on adverse events reported.

Results

One hundred and forty patients received IV levonadifloxacin therapy, 76 patients received oral alalevonadifloxacin, and 11 received IV followed by oral therapy. The mean duration of therapy was 7.3 days. Out of 227 patients, MRSA isolates were identified in 79 patients. Clinical success rates with oral, IV, and IV followed by oral levonadifloxacin therapy were 97.3%, 97.8%, and 100% respectively. The overall microbial success rate was 99.2% and only two patients reported two adverse events.

Conclusions

The excellent safety and efficacy profile of levonadifloxacin on oral and/or intravenous therapy, makes it a desirable treatment modality for management of ABSSSI. Unique features of levonadifloxacin such as availability of both IV and oral form, minimal drug-drug interactions, exemption from dosage adjustment in renal and hepatic impaired patients and a broad spectrum of coverage, makes it a suitable agent meeting several unmet clinical needs in contemporary patients.

## Introduction

Acute bacterial skin and skin structure infections (ABSSSI) are common in patients with damaged and compromised skin integrity [1]. Other predisposing factors for ABSSSI include skin conditions like tinea pedis, chronic skin lesions, and edema due to venous insufficiency [2]. The US Food and Drug Administration definition of ABSSSI includes cellulitis/erysipelas, major skin abscesses, and wound infections, with a lesion surface area of >75 cm^2^ (measured by the area of redness, edema, or induration) [3]. Skin and skin structure infections (SSTIs) are responsible for approximately 10% of all hospital admissions for infections across the globe [4]. A rising number of SSTI outbreaks globally leads to a significant increase in morbidity, mortality, and high direct and indirect costs. Gram-positive bacteria commonly cause skin infections, and significant proportions of them are caused by methicillin-resistant Staphylococcus aureus (MRSA) [5]. S. aureus, including MRSA, is commonly isolated in the skin, soft-tissue infections, and systemic infections involving the respiratory tract. Since the early 1990s, epidemics of skin infections have been associated with the hospital- and community-acquired MRSA [6].

MRSA infections are a serious healthcare problem, accounting for 30% to 70% of all S. aureus infections, and are of high global importance as a cause of community-acquired and healthcare-associated infection [7]. In India, high rates of MRSA have been reported among the S. aureus pool in clinical isolates from various studies, with rates as high as 54.8% (range, 32% to 80%) [8]. Despite the availability of various antimicrobial agents (AMAs), the intensive care unit (ICU) mortality in patients with multidrug-resistant Gram-positive infections (GPIs) is up to 16% [9].

Treatment of ABSSSI is challenging, as treatment choice is often empirical with varying doses and duration of therapy. Also, due to the limited efficacy, higher antimicrobial resistance, associated toxicity, and other limitations of existing AMAs, there is a need for a highly effective and safe drug to treat MRSA-associated ABSSSI. Despite the need, no new anti-MRSA antibiotics with IV and oral formulations have been introduced in India over the last 15 years. The complexity of treating ABSSSI has increased as patients often present themselves with comorbidities such as diabetes, obesity, and renal and hepatic dysfunction. Moreover, with advancements in medical practice, patients expect early clinical benefits in less severe skin infections, which requires drugs with rapid bactericidal action, excellent pharmacokinetics and pharmacodynamics (PK/PD) profile, and good coverage of multidrug-resistant S. aureus with an oral option. The management of such patients could be significantly improved with a drug that does not require dose adjustments or on-therapy monitoring of safety parameters.

Levonadifloxacin and its oral prodrug, alalevonadifloxacin, comprise a novel benzoquinolizine subclass of quinolone derivatives. These drugs have unique multispectrum antimicrobial coverage, including clinically significant Gram-positive and Gram-negative, atypical, and anaerobic pathogens [1]. Levonadifloxacin is indicated in ABSSSI, diabetic foot ulcers, and concurrent bacteremia. It has shown excellent activity against Gram-positive organisms like S. aureus (including methicillin-resistant and quinolone-resistant isolates), Streptococcus pyogenes, and Enterococcus faecalis (including vancomycin-resistant isolates), and Streptococcus dysgalactiae spp. dysgalactiae. Its potent activity is attributed to the unique mechanism of action that primarily inhibits DNA gyrase [10]. Levonadifloxacin has resistance suppression features as it is not a substrate for the NorA efflux pump, and it has low mutant prevention concentration, leading to a narrow mutant selection window. It also maintains bactericidal activity against a high bacterial load. Further, it has enhanced activity in an acidic intracellular environment and against biofilms. Therefore, it emerges as a safe and effective therapy in treating difficult-to-treat MRSA infections [11].

Moreover, due to higher intracellular concentrations in different tissues, levonadifloxacin is a well-suited AMA for treating skin and soft tissue infections caused by both Gram-positive and Gram-negative bacteria [10]. Levonadifloxacin also has an excellent safety profile, making it a better option for treating various bacterial infections. Therefore, we conducted this study to measure the outcomes of treating ABSSSI patients with levonadifloxacin as oral, IV, or combination oral and IV therapy.

## Materials and methods

Patients and methods setting

This data is a part of a post-marketing, multi-centric, retrospective, observational study (PIONEER study) conducted to assess the safety and efficacy of levonadifloxacin against various bacterial infections in real-world settings. We report the outcomes of levonadifloxacin used empirically in treating patients with ABSSSI from 84 hospitals across India from July 2020 to March 2021.

Informed consent and ethics

As a part of the retrospective observational study, we collected the prescription data of 227 patients receiving treatment for ABSSSI from 84 participating sites. The Institutional Ethics Committee (IEC) of D Y Patil University School of Medicine, Navi Mumbai (DYP/IEC/06- 019/2020) reviewed and approved the study-related documents. The study followed the principles of the Declaration of Helsinki (World Medical Association) and Good Clinical Practice guidelines issued by the Indian Council of Medical Research and the Central Drugs Standard Control Organisation of the Government of India. This study was registered with the clinical trials registry of India (CTRI/2020/09/028152). As this was a retrospective study, patient consent was obtained wherever possible, and strict confidentiality was maintained regarding patient identity.

Study participants

Data of 227 patients of any gender older than 18 years of age who received empirical levonadifloxacin (oral or IV) were captured in the study. The clinical diagnosis of the ABSSSI was based on the clinical and microbial test results. A study-specific data capture tool was used for data collection from the participating sites. Data included the patient's clinical condition on admission, associated comorbidities and complications, and concomitant therapy (including AMAs). Microbiological data were recorded wherever available. Patient management was at the discretion of the treating investigator.

Study outcomes

The primary outcomes were a clinical success and microbial success at the end of therapy. Clinical success was defined as resolution or improvement in signs and symptoms without further antibiotics. Clinical failure was defined as persistence or worsening of signs/symptoms, the need for additional AMAs, the occurrence of a new infection, or death. Microbial success was defined as the absence of organisms in the follow-up microbial testing in those patients where organisms were detected at baseline or negative culture during follow-up microbial testing.

Treatment safety was evaluated based on the clinical and laboratory adverse events. The treating investigator used a five-point Likert scale (with scores of excellent, very good, good, satisfactory, and poor) to assess the efficacy and safety of each patient.

Statistical analysis

This being a retrospective study without any hypotheses, data is presented as descriptive. The data were entered into a Microsoft Excel Worksheet. Descriptive analyses are presented for demographics and study outcomes. Measurement data are presented as mean and standard deviation (SD), and categorical data are presented as percentages.

## Results

Patient characteristics and pretreatment data

The study included 227 patients with a median age of 59.0 years. Most participants were men (n=155, 68.3%) and 72 were women (31.7%). Table 1 presents the demographic data and duration of levonadifloxacin therapy. Most of the patients (n=140; 61.7%) received IV levonadifloxacin, 76 patients (33.5%) received oral therapy, and 11 patients (4.8%) received IV therapy followed by oral levonadifloxacin. Of the 227 patients, 177 were hospitalized (78.0%), 50 patients (22.0%) were treated in an outpatient setting. The most common comorbid conditions encountered were diabetes (17.2%) and hypertension (10.6%). Other comorbidities were renal disorders (1.8%), thyroid disorders (1.8%), ischemic heart disease (1.3%), malignancy (1.3%), and hepatic disorders (1.3%). Of the 227 patients, 82 (36.12%) had cellulitis, 10 (4.4%) had infected wounds, four (1.8%) had an abscess, two (0.9%) had infected burns wound, and levonadifloxacin was used as surgical prophylaxis in six (2.6%) patients.

**Table 1:** Demographic data and duration of levonadifloxacin therapy

Levonadifloxacin		Age in years	BMI in kg/m^2^	Duration of therapy (days)
IV (n=140)	Mean	57	25.17	7
	SD	12.8	3.92	2.06
	Median	58	25.09	7
	Range	21 - 81	15.43 - 38.10	3 - 15
Oral (n=76)	Mean	57	25.75	8
	SD	13.3	3.75	2.85
	Median	59	25.23	7
	Range	24 - 85	15.78 - 36.79	3 - 15
IV followed by oral (n=11)	Mean	57	25.74	10
	SD	19.9	4.13	3.31
	Median	59	24.91	9.5
	Range	21 - 85	20.03 - 34.01	5 - 16
All patients (n=227)	Mean	57	25.39	7
	SD	13.3	3.87	2.48
	Median	59	25.10	7
	Range	21 - 85	15.43 - 38.10	3 - 16

Table 2 presents the organ systems involved in bacterial infection and existing complications in patients treated with levonadifloxacin. Various infection complications were reported in 88 (42.9%) patients, with renal impairment being the most common (20.3%). Other complications included septic shock (9.3%), systemic inflammatory response syndrome (7.9%), multi-organ failure (7.5%), hepatic impairment (7.0%), and thrombocytopenia (5.3%).

**Table 2:** Preexisting complications at time of admission for ABSSSI (n=227)

Preexisting Complications	Frequency, n (%)
SIRS	18 (7.9%)
Septic shock	21 (9.3%)
Multiorgan Failure	17 (7.5%)
Renal impairment	46 (20.3%)
Hepatic Impairment	16 (7.0%)
Thrombocytopenia	12 (5.3%)
Other complications	13 (5.7%)
Total complications	88 (42.9%)

Of 227 patients, microbial testing was conducted in 133 patients. Gram-positive organisms like S. aureus were the most common (41.9%), including MRSA (34.8%), followed by Gram-negative organisms (16.3%). Mixed infections were observed in 11.5% of patients and anaerobic organisms in 7% of patients. Table 3 presents the microbial testing results.

**Table 3:** Microbial testing results at baseline (n=227)

Organisms detected	Frequency, n (%)
Gram-positive	95 (41.9%)
Gram-negative	37 (16.3%)
Atypical organisms	14 (6.1%)
Anaerobic organisms	16 (7.0%)
Mixed infections	26 (11.5%)

Levonadifloxacin monotherapy was used in 37.9% (n=86) patients, and additional AMA was used in 62.1% (141) patients. Only two patients were prescribed two AMAs along with levonadifloxacin. Meropenem, imipenem, and carbapenem were the most commonly coprescribed AMA in 33.0% of patients, followed by beta-lactam antibiotics (14.5%); 45 patients (19.8%) received some other AMA. Other concomitant drugs were oral antihyperglycemic agents (9.3%), angiotensin-converting enzyme inhibitors/angiotensin II receptor blockers (4.8%), antiplatelet drugs (1.8%), beta-adrenergic blockers (0.9%), insulin (1.8%), statins (0.4%), heparin (0.9%), and other drugs (10.1%).

Clinical and microbial success

The overall clinical success rate with IV levonadifloxacin was 97.8%, with oral alalevonadifloxacin at 97.3% and a combination of IV followed by oral therapy had 100% clinical success (Table 4). Data from 132 patients were available to assess microbial success where bacterial cultures post-treatment were compared to baseline values. The microbial success rate was 99.2% (132/133), with microbial failure in only one patient who did not show clinical improvement.

**Table 4:** Clinical and microbial outcome with levonadifloxacin therapy

Levonadifloxacin treatment	Clinical outcome		Microbial outcome	
	Total	Success, n (%)	Total	Success, n (%)
IV	140	137 (97.8%)	104	103 (99%)
Oral	76	74 (97.3%)	25	25 (100%)
IV followed by oral	11	11 (100.0%)	4	4 (100%)
Total	227	222 (97.8%)	133	132 (99.2%)

**Figure 1:**
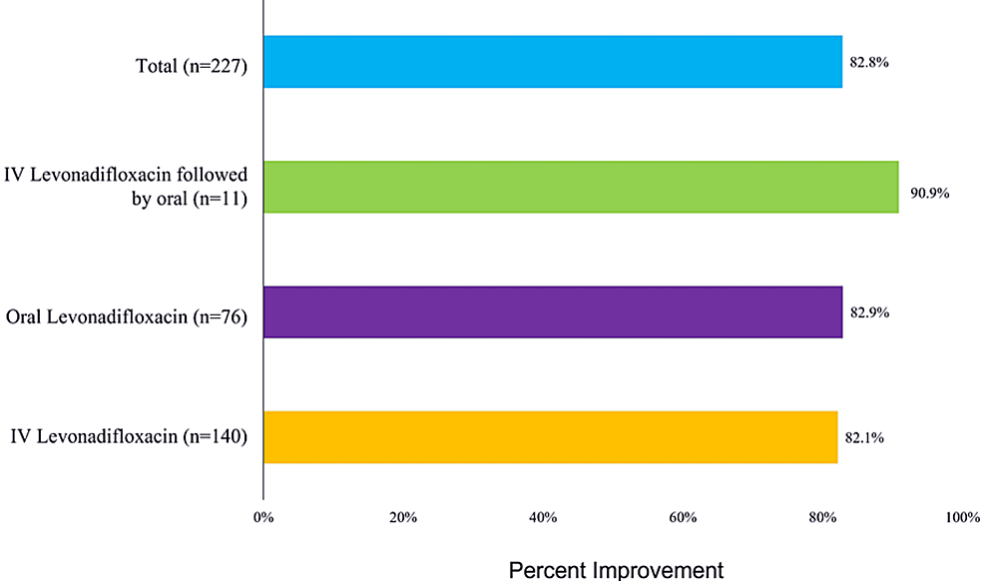
Clinical improvement Day 4

Most patients i.e., 82% patients showed clinical improvement in 72 to 96 hours (Figure 1). The mean duration of therapy with levonadifloxacin was 7.19 days with IV therapy, 7.63 days with oral therapy, and 9.6 days for IV followed by oral therapy.

Global assessments

**Figure 2:**
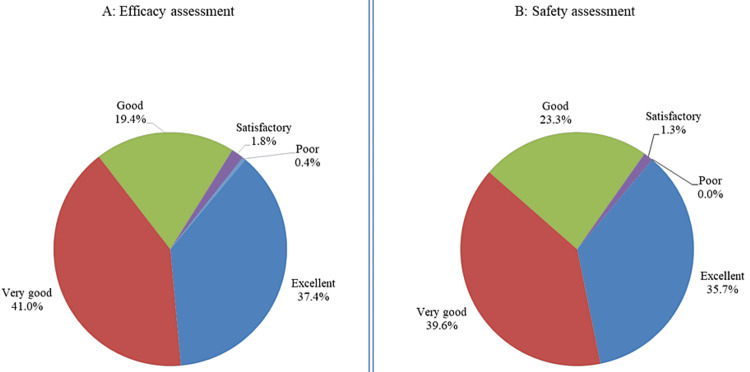
(A, B) Global assessment at the end of therapy (%, n=227)

Figure 2 presents the global assessments for efficacy (Figure 2A) and safety (Figure 2B) at the end of therapy. Overall, investigators rated the global efficacy as “good to excellent” in 97.8% of patients and “satisfactory” in 1.8% of patients. For global safety, investigators rated the safety as “good to excellent” in 98.7% of patients and “satisfactory” in 1.3% of patients.

Safety

Of 227 patients, only two adverse events were reported in two patients. One patient on IV therapy reported diarrhea, and one on oral therapy reported vomiting. Both events were of mild severity and resolved subsequently. There were no serious adverse events reported in the patient records.

## Discussion

In this study, we assessed data from 227 patients on oral and IV levonadifloxacin from all regions of India and documented real-world outcomes of levonadifloxacin for treating ABSSSI. Patients were prescribed levonadifloxacin for various types of skin and soft tissue infections, including those caused by MRSA infection.

A variety of medications have been used to treat MRSA in the past, with varying efficacy and risk profiles. Vancomycin has been the preferred drug for MRSA infections for many decades. However, the dose needs to be modified in patients with compromised renal function and higher vancomycin doses may be associated with increased nephrotoxicity and high-frequency hearing loss in older patients [12]. It is also associated with minimum inhibitory concentration (MIC) creep. Teicoplanin is a bactericidal agent but requires 48 to 72 hours to achieve optimum therapeutic concentration despite administrating a loading dose, has unpredictable tissue penetration, needs dose modification in renal impaired patients, may cause nephrotoxicity, and is also associated with MIC creep [1]. Daptomycin disrupts the bacterial cell membrane, leading to rapid depolarization and cell death. However, it is inactivated by a pulmonary surfactant and has skeletal muscle toxicity. It is unsuitable for widespread use as it requires dose adjustment in renal impaired patients. All such treatments require on-therapy monitoring of safety parameters that adds to the burden on hospital staff in terms of constant vigil for safety signals which increases the cost of treatment. However, linezolid is bacteriostatic against S. aureus but may cause peripheral neuropathy, myelosuppression, and lactic acidosis in long-term use [1]. Similarly, tigecycline is bacteriostatic and has poor tissue penetration. It has a black box warning about the risk of death with IV use [13].

A survey of the trends in the management of GPIs in Indian critical care settings highlighted an increasing trend in prevalence and associated mortality with GPIs [12]. To address the challenge of growing multidrug-resistant pathogens and limitations associated with existing anti-MRSA agents, there is a need for a new suitable empirical agent with an improved safety profile, especially in the ICU [9]. The Infectious Disease Society of America guidelines emphasizes adequately controlling the source of infection, such as draining abscesses and debridement of nonviable tissues irrespective of the AMAs used [13]. Thus, additional antimicrobial options for ABSSSI are essential for combating resistance to existing therapies and improving patient outcomes.

S. aureus, a facultative intracellular pathogen, is a common pathogen responsible for both community- and hospital-acquired infections [14]. As reported in Ghia et al.'s meta-analysis, the treatment options identified for MRSA in India include arbekacin sulfate, vancomycin hydrochloride, teicoplanin, daptomycin, and oritavancin [15]. However, many anti-staphylococcal drugs' lack of intracellular activity could be responsible for persistent S. aureus infections such as persistent bacteremia and failure of therapy. Although several treatment options for ABSSSI are available, including daptomycin, linezolid, tigecycline, teicoplanin, and vancomycin, treatment is becoming more difficult due to increasing antibiotic resistance [2].

Levonadifloxacin and its ester oral prodrug, alalevonadifloxacin, are novel broad-spectrum antibiotics with coverage of multidrug-resistant Gram-positive pathogens including MRSA, heterogeneous vancomycin-intermediate S. aureus (h-VISA), and vancomycin-resistant S. aureus (VRSA), as well as quinolone-resistant strains [16]. Levonadifloxacin has potent killing action against both the methicillin-susceptible S. aureus and MRSA in an intracellular environment of THP-1 macrophages [17,18]. The intracellular activity of levonadifloxacin was better than that of other fluoroquinolones (moxifloxacin and ciprofloxacin) [19]. Thus, due to its high intracellular activity, both oral and IV levonadifloxacin can offer a better therapeutic option for treating persistent MRSA infections than previous options [17]. Levonadifloxacin has been shown to exhibit potent in-vitro activity against contemporary S. aureus isolates (including the Bengal Bay clone) collected from a large Indian tertiary care hospital, where 793 isolates were studied [20]. For all S. aureus strains, Levonadifloxacin showed MIC50 of 0.25 mg/L and MIC90 of 0.5 mg/L. MIC50 and MIC90 values are the lowest concentration of the antibiotic at which 50% and 90% of the isolates were inhibited. Levonadifloxacin has shown to retain good potency against strains with multiple mutations in S. aureus in quinolone targets [10].

Most patients in our study (65.1%) had GPIs. With oral and IV levonadifloxacin, we observed clinical success rates of 97.3% and 97.8%, respectively, for the treatment of ABSSSI. Additionally, a high systemic exposure along with high drug levels in tissues indicates the clinical utility of levonadifloxacin for the treatment of ABSSSI caused by extracellular as well as intracellular pathogens [12]. Our study included patients with mixed infections and possibly Gram-negative bacterial infections (25.3%) and anaerobic organisms (11.0%). To treat polymicrobial infections, levonadifloxacin offers a simplified therapeutic advantage as it has activity against Gram-negative, atypical bacteria and anaerobes [21]. In our study, 17.8% of patients (n=26) had polymicrobial infections, and the clinical success rates were more than 90.0%.

The results of a Phase 3 study of levonadifloxacin versus linezolid in 501 patients with ABSSSI were recently published [22]. The clinical cure rates observed in the modified intent-to-treat populations for levonadifloxacin were 91.0% with IV treatment and 95.2% with oral treatment. The authors concluded that both oral and IV levonadifloxacin are noninferior to linezolid for the treatment of ABSSSI. Another Phase 3 study compared IV (300 mg) followed by oral delafloxacin (450 mg) versus vancomycin (15 mg/kg IV) plus aztreonam for five to 14 days included 850 adults with ABSSSI [23]. The fluoroquinolone therapy was noninferior to IV vancomycin plus aztreonam combination therapy for both the objective response and the investigator-assessed response at initial and subsequent follow-up. The objective response was 83.7% in the delafloxacin arm and 80.6% in the vancomycin plus aztreonam arm. However, treatment-emergent adverse events leading to study drug discontinuation occurred in only 1.2% of patients with delafloxacin and 2.4% with vancomycin plus aztreonam. There was no discontinuation of levonadifloxacin therapy in our study due to adverse effects. In an integrated analysis of two randomized control trials (Omadacycline in Acute Skin and Skin Structure Infections Study; OASIS-1 and OASIS-2), omadacycline, a novel aminomethylcycline, was compared with linezolid to treat ABSSSI [24]. Omadacycline was noninferior to linezolid with an early clinical response of 86.2% vs. 83.9%. Clinical responses were similar in different infection types. However, the treatment-emergent adverse events were reported by 51.1% of patients receiving omadacycline and 41.2% of those receiving linezolid. Similar clinical response rates are observed with linezolid 79.0% and tedizolid (87.1%) in another outpatient study of ABSSSI treatment in the USA [25].

We observed an overall clinical success rate of 97.8% with levonadifloxacin therapy (oral/IV) for treatment of ABSSSI, which seems to be equivalent to responses to most of the therapies for MRSA. It is noteworthy that the efficacy of levonadifloxacin is good despite many patients (42.9%) in our study having complications like systemic inflammatory response syndrome (SIRS), septic shock, multi-organ failure, renal impairment, hepatic impairment, and thrombocytopenia.

In our study, for most patients (97.8%), the global efficacy was reported as “good to excellent.” Multiple attributes of levonadifloxacin such as activity against atypical bacteria, potent activity of levonadifloxacin against anaerobic isolates (excluding Bacteroides fragilis, cidal activity against high-inoculum cultures, and slow-growing staphylococci), and improved activity in acidic pH, potentially contribute to its efficacy in treating ABSSSI, wound infections, and purulent conditions [21]. In addition, levonadifloxacin has excellent bioavailability of approximately 90% with oral formulation and a superimposable PK profile for both IV and oral forms. This allows smooth switchover from parenteral to oral therapy [12].

QT prolongation is a common risk associated with quinolones, with the highest risk associated with moxiﬂoxacin [26]. In a study involving 48 healthy subjects, a supratherapeutic dose (2600 mg) of levonadifloxacin was not associated with QT prolongation [27]. The safety of levonadifloxacin is well documented, and all studies published to date have no reported deaths, serious adverse events, or signiﬁcant abnormalities in clinical laboratory parameters, vital signs, 12-lead electrocardiogram changes, or ﬁndings on physical examination [28]. In our study, for most of the patients (98.7%), global safety was reported as “good to excellent.”

Our study had some limitations in being a prescription event monitoring study. Due to the observational study design, microbial testing results were not available for all patients at baseline and the end of therapy. Hence, limited data are available for the microbial success assessment.

## Conclusions

We aimed to capture real-world evidence from a retrospective observational study on the safety and efficacy of levonadifloxacin in the treatment of ABSSSI. Clinical improvement was seen in most patients within 72 to 96 hours with a mean treatment duration of seven days. The improvement may be attributed to levonadifloxacin’s enhanced activity in an acidic environment, rapid cidal action, and eradication of biofilms caused by resistant pathogens like MRSA, which are common in cases of ABSSSI. The safety and efficacy of levonadifloxacin assessed in this study make it a desirable treatment modality for the management of ABSSSI.
